# Using the Internet for Life Style Changes in Diet and Physical Activity: A Feasibility Study

**DOI:** 10.2196/jmir.6.3.e28

**Published:** 2004-09-08

**Authors:** Jacob Anhøj, Anne Holm Jensen

**Affiliations:** ^2^University of AarhusDepartment of Information and Media StudiesHelsingforsgade 14DK-8200 Århus NDenmark; ^1^AstraZeneca A/SAlbertslundDenmark

**Keywords:** Internet, life style, self care, physician-patient relations, computer-assisted decision making, user-computer interface

## Abstract

**Background:**

LinkMedica-Heart is a novel Internet based program intended to support people who seek to improve their life style by means of changes in diet and physical activity. The program is currently under evaluation in a clinical study and the present study is a feasibility test of the LinkMedica-Heart Internet based program.

**Objective:**

The aim of this study was to evaluate LinkMedica-Heart, an Internet based program we designed for support and maintenance of patient-led life style changes.

**Methods:**

The feasibility study of LinkMedica-Heart presented here is a qualitative study. Nine general practitioners were invited to participate. Each practitioner was asked to introduce LinkMedica-Heart to not less than two patients, with a maximum of five patients per practitioner. Patients and general practitioners were both asked to participate in testing the program for a period of 6 months. At the end of 6 months, evaluation meetings were held with the general practitioners, and separate interviews took place with some of the participating patients who were selected by the GPs.

**Results:**

Five general practitioners and 25 patients participated in the study. The general practitioners and the patients were enthusiastic about the prospect of an Internet based life style change program. However, the program was not able to sustain patient loyalty over an extended period. The doctors found that the program was much too complicated to navigate and that the results from the program could not be trusted. The patients in contrast had fewer complaints about the program design, but found that the advice given by the program was too elaborate and detailed and, in general, did not add to the patient's knowledge on life style change.

**Conclusion:**

Our study confirms that there is a need for, and a receptive attitude toward a Web-based program that supports people who want to improve their life style and health. LinkMedica-Heart in its present form does not satisfy these needs. We suggest a number of design changes and improvements to the program.

## Introduction

Obesity and physical inactivity are major causes of a number of diseases. Several studies prove that a change of life style towards a healthy diet and ample physical activity reduces the risk of a large number of diseases [1,2]. However, permanent life style changes are difficult to achieve for most people.

Individual and group based education of individuals for implementing life style changes are seldom possible due to often-limited resources. There is an enormous need for innovative ways to introduce and maintain life style changes in people at risk from life style related diseases.

The growth and extensive use of the World Wide Web presents a novel opportunity for mass communication and patient education (also referred to as e-learning). Taking advantage of the new medium, the Research Centre for Prevention and Health (RCP), Glostrup, Denmark, in cooperation with AstraZeneca (AZ), Denmark, created a novel Internet-based program called LinkMedica-Heart (LMH), for support and maintenance of life style changes in people at risk from life style related diseases. The program's effect on predictors of life style related diseases is currently under investigation in a clinical trial that compares changes in serum cholesterol in patients using the LMH program, with those of patients using a ‘placebo' program. We plan further studies on other predictors of life style related diseases that may be prevented with the LMH program.

The present study was originally intended to be a pilot test with a small number of general practitioners (GPs) testing the web pages, together with some of the practitioners' patients, in order to help us identify and correct errors in the program prior to beginning the actual clinical study mentioned above. The feedback we got from the GPs and some of the patients during the pilot study could be of interest for other researchers working on Internet-based patient education. Hence, we decided to expand the pilot study with some in-depth interviews with selected patients and present our findings on the feasibility of LinkMedica-Heart as an Internet-based program for support and maintenance of life style changes.

## Methods

The aim of LinkMedica-Heart is to provide support and information for people trying to achieve a healthy life style through focus on diet and physical exercise. LMH is part of a closed Web site that is currently accessible only by people who participate in the study.

### System Description

**Figure 1:**
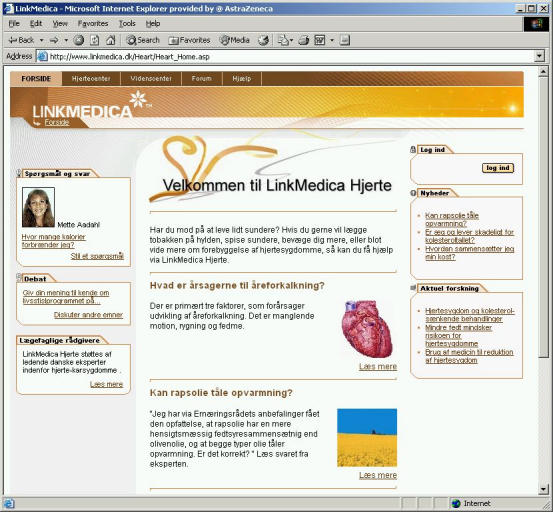
LinkMedica-Heart home page

To use LMH, the patient must register and chose a username and a password. The patient is then required to enter his or her sex, height, weight, and E-mail address. A screenshot of LMH's home page is shown in Figure 1.

**Figure 2:**
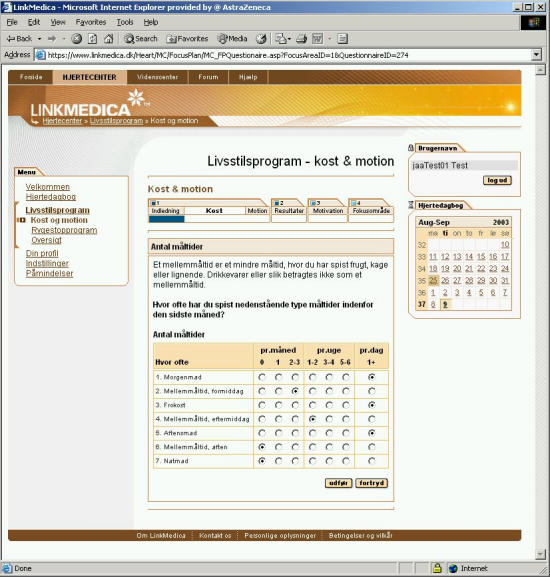
A page from the diet questionnaire. Each question, eg, “Morgenmad” (Breakfast) or “Frokost”(Lunch), requires patients to answer how often they have consumed an item during the previous month. Other questionnaire pages detail contents of each type of meal

**Figure 3:**
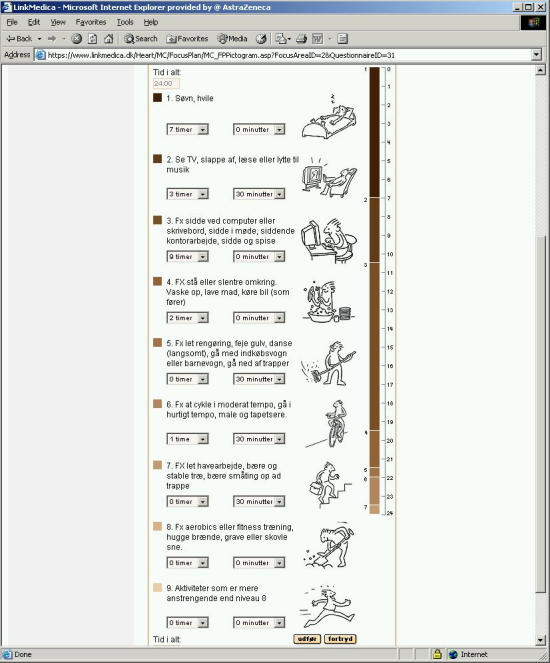
Screen-shot of the physical activity questionnaire. A cartoon accompanying each question illustrates the activity level. The patient needs to answer with the average time spent per day, in hours and minutes, on an activity level. The answers must total 24 hours

After registration, the user is asked to activate a life style change program. To do this, the patient must first fill-in two questionnaires: one about diet, and the other about physical activity (screenshots shown in Figure 2 and Figure 3).

The diet questionnaire is very detailed and asks the patient how often he or she has consumed different food items typical in a Danish diet in the last month. The LMH program then matches each answer with data stored in the program's database on the nutritional composition of the particular food item entered by the patient. The system assumes the size of an average serving of the particular food item and calculates the patient's intake of energy, fat, protein, carbohydrates, fibre, and alcohol. In total, there are 219 questions, and filling in the diet questionnaire for the first time takes approximately half an hour. The diet questionnaire is based on the Dankost 2000 computer program [3].

In the activity questionnaire, the patient must specify on average how much time he or she spends, during a typical day, on nine different activity levels that extend from sleep to hard physical activity. The total time must add up to 24 hours. The LMH program assigns each activity level a metabolic equivalent (ME) number. One ME corresponds to a person's energy expenditure when sitting relaxed. The ME of an activity times the patient's weight, the minutes spent per day on the particular activity, and a constant for the patient's sex, gives the amount of energy spent on the particular activity, per day. The total energy spent per day is a sum of energy spent on all activities in a day. The activity questionnaire takes only a few minutes to fill-in.

**Figure 4:**
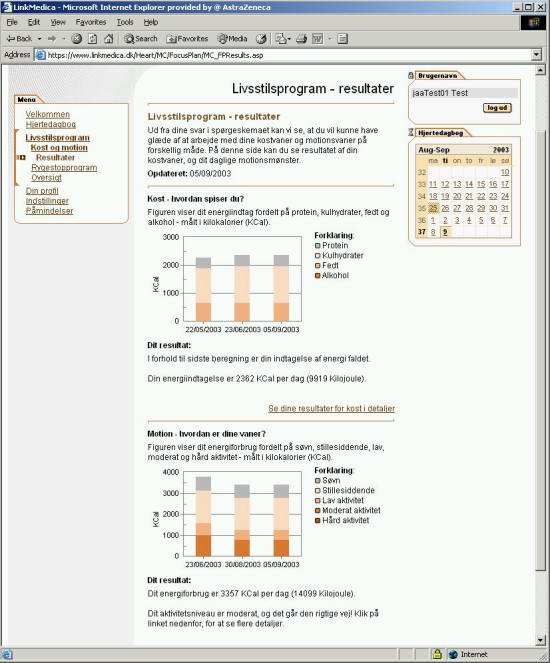
Result page. The two graphs show the daily energy intake and energy outflow of the patient. Each colour in the bars indicates a constituent of the diet, eg, protein, carbohydrate,fat, and alcohol, or an activity, eg, sleeping, sitting, low, moderate, and vigorous physical activity.

After filling-in both the diet and activity level questionnaire, the patient is presented with results that summarize energy intake, energy expenditure, and composition of diet with regard to protein, carbohydrate, fat, alcohol and fibre per day (Figure 4).

The patient is then asked to select one of five offered diet programs, and one of three activity programs. The patient can select a program based on how motivated the patient is for a life style change: a highly motivated patient might select an intensive program, while a less motivated patient may select a program that aims at creating the needed motivation.

After the patient has activated a program, he or she receives a computer-generated E-mail with results of the questionnaire and personalized advice on how to improve life style through changes in diet and physical activity. The activity questionnaire, the advice generator, and textual content were created by RCP.

After 4 weeks, patients received an E-mail asking them to update answers to the diet and physical activity questionnaires. When updating their answers, patients were only required to enter changes in diet or physical activity since the last questionnaire. This made updating much less demanding than the first entry. Again, the patient received results and advice based on the current life style change program. The patient could at any time change their program to a more or less intensive one. The content of E-mail advice changed according to the newly recommended program. Thus, the overall goal of the program was to introduce and maintain permanent changes in life style, rather than a radical short-term change in diet.

A single life style change program ran for 6 months. If the patient chose a different program in between, the new program would then take over for the next 6 months.

### Data Collection

Nine GPs, selected by two AstraZeneca sales representatives, were invited to participate in the feasibility test of LMH. Each GP was asked to introduce between two to five patients to the LMH program. During the feasibility test, the GPs were asked to see each patient at least once after 3 to 6 months after the patient was introduced to the program. After 6 months, we held an evaluation meeting with the GPs. At this meeting, the GPs gave an oral summary of their own and their patients' experience with LMH. Two persons prepared separate minutes of these meetings.

We further asked two of the GPs to select a total of four patients who had participated in the feasibility test to participate in semi-structured in-depth interviews. We chose to interview patients as a qualitative research method, since the feasibility of the program could not be quantified or analysed by statistical methods. Qualitative research focuses on people's lives, their experiences, and emotions, as well as cultural phenomena and social movements [4].

One of the methods used in qualitative research is the semi-structured interview, the main source of empirical material in this study. According to Kvale, the purpose of the qualitative research interview is to describe and interpret themes in the patients' lifestyle that shape a continuum between description and interpretation [5].

In this study, the main research questions for which we sought answers in the patient interviews were: (a) how do patients use the lifestyle program, and (b) what do they learn about lifestyle when interacting with the program. To answer these questions, we made an interview guide which outlines three main themes for the patient interview sessions: the patients' (a) extent of involvement in the pilot study, (b) use of the LMH lifestyle change program, and (c) extent of putting into practice advice given by the program.

Interviews were held by the second author in the patients' homes to help make them feel comfortable during the interview sessions. In addition, a student of psychology was present as an observer.

The interviews lasted between 45 and 60 minutes and were recorded on Minidisks. Immediately after each interview, the interviewer and the observer discussed their interpretations of the interview, and the patient's attitude towards the program. Later, the interviewer alone revised the interview recordings three times: the first was a general recall of the interview without a specific focus; the second time, the interviews were divided into sections by means of the Minidisk player's bookmark function. Each interview had at least 30 to 40 sections. From each section, significant words and statements were noted and used in the third revision to organize each interview into thematic units.

We also received some unsolicited E-mails and telephone calls from patients who participated in the study, conveying their experience with the program. In addition, one GP asked his patients to fill-in a questionnaire regarding four aspects about LMH: usability, reliability, suggestions for improvement, and relevance of the program for lifestyle change.

The minutes of the interviews, together with the E-mails, questionnaires and calls from patients comprise the data material of this study.

## Results

Three GPs withdrew from the pilot test, one because of illness, and two because of time constraints. One GP was unable to get any patients signed up for the study due to technical problems with his Internet connection. Another GP could not participate in the evaluation meeting after the pilot test and sent his comments by E-mail. Table 1 summarizes the demography of the five GPs who participated in the study. The five GPs introduced a total of 25 patients (including 13 females and 12 males), between the ages 23 to 80 years (median age = 43 years) to the LMH pilot study.

Four patients were interviewed for the qualitative research interview. Table 2 profiles the patients interviewed.

Additionally, we received two unsolicited E-mails from two participating patients, and four anonymous filled-in questionnaires turned-in by patients of the GP who could not attend the evaluation meeting.

**Table 1:** Demography of general practitioners

**General Practitioner (GP)**	**Description**
GP1 (Jakob Dahl)	Male, 39 years of age, 5 years in general practice, urban area, 2 doctors in clinic
GP2 (Lars Dudal)	Male, 50 years of age, 13 years in general practice, rural area, 2 doctors in clinic
GP3 (Henning Skytte)	Male, 51 years of age, 17 years in general practice, rural area, 5 doctors in clinic
GP4 (Dennis Christoffersen)	Male, 47 years of age, 7 years in general practice, urban area, 4 doctors in clinic
GP5 (Jesper Holmelund)	Male, 47 years of age, 8 years in general practice, rural area, 3 doctors in clinic

**Table 2:** Demography of patient interviewees

**Patient**	**Description**
A	Female, 44 years old, married, 2 adult children, social worker, overweight
B	Male, 55 years old, married, 2 adult children, former bank employee, currently unemployed, diabetes, hypertension, hypercholesterolemia
C	Female, 43 years old, married, 2 children (1 adult), nursing aide, overweight
D	Male, 40 years old, married, 2 children, veterinary aide, overweight, hypertension

### Results From Evaluation Meeting With GPs

Four topics came-up during discussions at the evaluation meeting:

- Program errors
- Initial perception of the program concept
- Program usability
- Program design.

#### Program Errors

Early on during patient inclusion in the feasibility test, we discovered that the E-mail service did not work, and advice and reminder E-mails were not mailed to patients. This error affected about half the patients for a month and a half, and clearly gave them the impression that the program was ‘dead'.

A small number of minor errors, primarily in algorithms controlling advice messages were also found and corrected.

#### Initial Perception of the Program Concept

Before they were introduced to the LMH program, the GPs, their nurses, and patients were enthusiastic about the idea of having a life style program available on the Internet. The GPs agreed that general practice needs new means to help introduce and maintain life style changes for patients at risk of life style related diseases. One GP (GP2) said, “The patients were happy when I asked them to participate in a test of a life style program. When I introduced the program to them, their eyes shined and they were ready to start immediately.”

An anonymous letter from a patient noted, “Unfortunately, I never got into the program, but I wanted to do it very much. I believe I would have benefited from it.”

However, during the feasibility test it became obvious from informal contacts (telephone conversations and E-mails) that most patients perceived the program as a *short-term* *diet* program, rather than a program intended for long-term *permanent* changes in life style. For example, a middle-aged female expressing her expectations in an E-mail said: “It would have been great if you had to enter your diet every day instead of only once a month. This is important when you have to lose weight. In this way you get the feedback immediately and can correct your mistakes the day after.” We were surprised by this error in perception, since we had worked very hard to communicate LMH as a lifestyle change program rather than a diet reduction program during initial meetings with GPs and on the LMH Web site.

#### Program Usability

As expected, the GPs had different opinions regarding the LMH Web site layout: one GP found the site layout elegant; another GP found it boring with tiny fonts and too few graphics; and the other three GPs had no special opinion for or against the layout.

All GPs, however, agreed that the program was much too complicated to navigate. GP3 said, “The program is complicated, and navigation is confusing. It is difficult to navigate the site.” GP2 stated, “When a patient logs on for the first time, the program should be extremely simple with the possibility of adding more and more functionality as the patient gains experience.”

Interestingly, some patients did not agree with the GPs negative perception of the site's usability. The same patient who expressed her perception of the program as a diet-reduction program wrote in her E-mail, “First I want to say that the site is logically built, easy to navigate and has some very good information.”

#### Program Design

The term ‘program design' refers to the logical design of the LMH program—from the patient when first filling-in the questionnaires, viewing the results, then choosing a life style change program in keeping with the extent of personal motivation, and finally, receiving regular advice by E-mail.

The GPs agreed that the questionnaires, especially the diet questionnaire, were far too long. GP3 said, “The questionnaires are too long, and the patients lost interest very quickly.” The GPs also had doubts about the reliability of the results. As GP1 said, “The patients did not trust the results.” This view was supported by GP2 who tried the program himself and found that the results did not match his own observations on the balance between his energy intake and expenditure.

In general, there were surprisingly few comments on advice given via E-mail. This, of course, was partly due to the program error that withheld E-mails to patients for a month and a half. However, the advice was also available on-line on the LMH Web site and the patients did not seem to pay much attention to the available advice. GP3 told us, “Most patients said that they had not seen the advice content and if they had, they did not trust them because they were in conflict with their personal experience.”

An important observation from the study is that none of the patients completed their life style change program. Personal questionnaires were updated no more than a few times, if ever. The patients did not give any clear explanation why they lost interest in the program. They simply disclosed that they stopped using the program. In an anonymous questionnaire a patient wrote: “To be honest, I have not entered the site more than once.”

The GPs, too, agreed that the program was unable to sustain the patient's attention for more than a short period. GP3 said, “… when using the program, they lost interest quickly.” GP2 summed up, “At the beginning, the patients were highly motivated. But the program is much too complicated.” In the GPs opinion, one reason for the patients' loss of interest was that the program's interaction with the patients was too infrequent and meagre. As GP1 said, “It is crucial to have frequent contact with the patients, especially in the beginning.”

### Results From Patient Interviews

From the analysis of patient interviews, four issues emerged:

- LMH did not provide patients with new information
- The feedback E-mail content was too detailed and elaborate
- Changes in life style do not come from using a computer based program alone
- Human support and contact are important if life style change programs are to succeed.

Although the content of advice based on the results from the questionnaires is highly detailed, the patients interviewed did not find that they were given any new information. This point became clear early in the interview sessions, as we noticed that the patients' knowledge of lifestyle and their general awareness of how to eat, drink, and exercise to stay healthy was high from the beginning. Consequently, the patients quickly lost interest the program since they felt that they already knew the outcome. Hence, the program was not able to establish a personal interaction with the patients. One of the patients, patient C, said, “I have loads of materials about healthy living. I don't need any more.” From the patients' viewpoint, the information on LMH is very basic and could be one of the reasons why the patients lost interest in the program. Patient A said in the beginning of the interview, “I have been on a diet since I was 11 years old, so I know a lot about what is healthy and what is not.” When another patient, patient D, was asked about his opinion of the program, his comment was, “I don't need the program to tell me that I eat less than I expend. I can see that on the scale – can't I?”

Regarding the texts being too broad and elaborate, patient D said that he spent too much time locating information on the LMH Web site and that he would rather call his doctor or nurse to get a quick answer. Another patient, patient B, preferred to have all information in print and said: “I spent 50 minutes printing everything, and I didn't even want to read it … it's too much. There is nothing wrong with the content, but it's too much.”

As for the third issue (i.e., changes in life style do not come from using a computer program alone), all patients agreed that the use of the program did not provide the necessary support in their struggle towards a healthy life style. They said that the program itself was merely a tool and that it could never replace support from a health care professional. One of the patients, patient C, noted that the computer could not keep an eye on her, as her nurse could, “I need another person to check my weight and say, ‘it's for your own good, it's not for me you are doing this.' The program may give me advice and ideas, but I need the nurse to coach me.”

Patients seemed to give a high value to personal relations with a health care professional. They expressed great conviction in the competence of the health care professionals, a trust that they could not seem to establish with the LMH computer based program. Hence, the patients were sceptical with the results that the LMH program provided. Patient A said, “… it's the doctor who is educated and knows things. You cannot expect a computer program to know what is good for you. However, if your doctor tells you to use a computer program, it might provide extra information.” As a result, patient A acknowledges that when the doctor endorses a program it must be of some value for her as a patient.

## Discussion

As stated in the introduction, this study was intended merely to be a test of the LMH program and to find software errors in the program. Data for the study was collected rather randomly from different sources: minutes from evaluation meetings with hand-picked GPs who reported their patients' experience with the LMH program; unsolicited E-mails and letters from patients; and issues appearing in interviews with selected patients. We acknowledge that the patients who where selected by the GPs to participate in the study, especially the patients selected for interviews, may not at all be a representative sample of the general population of people (not necessarily patients), needing life style changes. However, we believe that despite these limitations, our findings and conclusions may be of interest to other researchers working on internet based disease management or prevention.

Our pilot study confirms the need for, and an open attitude towards life style change programs delivered via the Internet. The doctors confirm the need to aid their patients starting a life style change program with a tool that complements the doctor's advice and direction. The patients, too, seem to be eager to get additional help and support in striving towards a healthy life. The Internet appears to be an ideal medium for providing the additional support that complements the doctor's counsel.

However, the present version of LinkMedica Heart does not seem to fulfil this need: the patients did not use the program more than a single, or a few times.

It is important to stress that the study was a real time study, with an aim to learn how GPs and their patients use and perceive LMH—after a relatively short interaction with the program. The objective was not to investigate the effect of the LMH program on patients using the program. Hence, we did not attempt to control either the GPs' or the patients' use of LMH during the test phase.

Our study identifies three issues that need to be addressed in order for the LMH program to be successful in bringing about patient lifestyle changes:

- Complicated user interface and navigation
- Reliability of questionnaire results and content of advice
- Lack of personal interaction between the program and the patient.

### Interface

It is tempting to think that if the user interface and navigation were improved, it would be sufficient to sustain the patients' attention. Without any doubt, an improved interface would certainly help encourage new patients to enter the program for the first time—especially if they are attempting to join the program on their own without support from a healthcare professional. However, improvements to the interface may not necessarily sustain participant loyalty to the program for an extended period. The patients included in the study were handpicked by their GPs and were cautioned that they were testing a new program with many rough edges. In addition, the patients in the study had support of their GP's to help them get started with the program. Hence, we do not believe that simply improving the user interface would be enough for LMH to be a successful Internet based program.

### Reliability

A lot of effort was put into creating the content and the logic behind the result and advice text for the LMH Web site. To make the results and advice precise and personal, a typical feedback message from LMH was almost as long as five printed pages. Nevertheless, some patients felt that the feedback was not in accordance with their own expectations. For example, the LMH program told some patients that their energy intake was *higher* than expenditure, when in fact they had been loosing weight. Some other patients were told that their intake was *lower* than their expenditure when in fact they were gaining weight. Moreover, patients were not inclined to read the lengthy textual content, either on the computer screen or as a print out of the text.

The accuracy of the energy calculations in the LMH program was not an issue in this study. However, accuracy of the calculations would be an issue and a major problem if patients were wary of the program overall. Any mistrust will inevitably lead the patients to stop using the program, even if it is the patient who is mistaken and not the program.

It might be a better idea to simplify the feedback function of the program engine with precise, qualitative advice in the form of concise messages based on a patient's own perception of his or her dietary habits, coupled with measured developments in weight and waist circumference. The questionnaires could also be similarly simplified.

The observation that patients are more likely to trust their own opinion than that of a computer program supports our previous findings from a similar Web service for asthma patients, LinkMedica-Asthma [6]. In the previous study, we found that, in general, asthma patients did not follow the advice given by the Web site, even if acknowledged asthma experts offered advice. When the advice was in disagreement with their own previous experience or attitudes, whether medically true or not, patients disregarded the advice

### Personal Interaction

The diet questionnaire is intended to reveal the diet for the previous month. Therefore, it would be misleading to ask the patient to update the questionnaire more often. On the other hand, the study shows that in order to sustain patient attention, the program needs to interact with patient more frequently, preferably on a daily basis. Some patients wanted to enter their questionnaires every day, while others preferred a less demanding program. Consequently, the ideal program should be adaptable to the patient's preference. Combining succinct, qualitative questionnaires with concise advice delivered via E-mail, and/or SMS (Short Message Service provided by wireless phone companies), could prove useful. Also, breaking up advice messages into small fragments, delivered on a daily basis might help patients who do not want to read long, all-inclusive texts.

### Suggestion to Improve the Site

In summary, we suggest a number of improvements to the LMH Web site:

- A simplified diet questionnaire for a *qualitative* evaluation of the patient's dietary habits.
- Frequent (preferably daily), and concise feedback messages from the program to the patient in the form of E-mail and/or SMS messages. These messages can possibly contain practical advice about healthy living, and food recipes attuned to the patient's lifestyle profile.
- Suggestions to monitor weight and waist circumference. Physical measures of weight and waist circumference can be the patient's means of assessing whether there is any positive effect of his or her lifestyle change efforts.
- Removal of options in choosing a lifestyle change program. The LMH program in its current state prompts the patient to chose between several lifestyle change programs based on his or her degree of motivation. Our study suggests that patients are highly motivated from the beginning and that too many program options confuse them. Hence, the program should automatically select the most appropriate lifestyle change program based on the patient's profile.

### Conclusion

This study confirms the need for, and a positive attitude towards Web-based programs for supporting people who want to improve their health through life style changes. In its present form, LinkMedica-Heart, our life style change program, does not address these needs. A number of design changes and improvements to the program are suggested.

## Abbreviations

GP: General Practitioner
HCP: Health Care Professional
SMS: Short Messaging System
LMH: LinkMedica-Heart
AZ: AstraZeneca
RCP: Research Centre for Prevention and Health
